# Phylogenetic Analysis of Brassica rapa MATH-Domain Proteins

**DOI:** 10.2174/1389202911314030007

**Published:** 2013-05

**Authors:** Liming Zhao, Yong Huang, Yan Hu, Xiaoli He, Wenhui Shen, Chunlin Liu, Ying Ruan

**Affiliations:** 1 1Hunan Provincial Key Laboratory of Crop Germplasm Innovation and Utilization, Hunan Agricultural University, Changsha, 410128 Hunan, China; 2 2College of Bioscience and Biotechnology, Hunan Agricultural University, Changsha, 410128 Hunan, China; 3 3Experimental Center of Hunan Agricultural University, Changsha, 410128 Hunan, China; 4 4Agricultural College of Hunan Agricultural University, Changsha, 410128 Hunan, China

**Keywords:** Brassica rapa, Phylogenetic analysis, MATH domain protein, Protein domain organization, Gene expression, Seed development.

## Abstract

The MATH (meprin and TRAF-C homology) domain is a fold of seven anti-parallel β-helices involved in protein-protein interaction. Here, we report the identification and characterization of 90 MATH-domain proteins from the *Brassica rapa* genome. By sequence analysis together with MATH-domain proteins from other species, the *B. rapa* MATH-domain proteins can be grouped into 6 classes. Class-I protein has one or several MATH domains without any other recognizable domain; Class-II protein contains a MATH domain together with a conserved BTB (Broad Complex, Tramtrack, and Bric-a-Brac ) domain; Class-III protein belongs to the MATH/Filament domain family; Class-IV protein contains a MATH domain frequently combined with some other domains; Class-V protein has a relative long sequence but contains only one MATH domain; Class-VI protein is characterized by the presence of Peptidase and UBQ (Ubiquitinylation) domains together with one MATH domain. As part of our study regarding seed development of *B. rapa*, six genes are screened by SSH (Suppression Subtractive Hybridization) and their expression levels are analyzed in combination with seed developmental stages, and expression patterns suggested that* Bra001786*, *Bra03578* and Bra036572 may be seed development specific genes, while *Bra001787*, *Bra020541* and *Bra040904* may be involved in seed and flower organ development. This study provides the first characterization of the MATH domain proteins in* B. rapa*

## INTRODUCTION

Meprins are mammalian tissue-specific metalloendopeptidases of the astacin family implicated in developmental, normal and pathological processes by hydrolysing a variety of proteins [1]. TRAF (TNF-receptor associated factors) proteins were first isolated through their ability to interact with TNF receptors [2]. The MATH domain (meprin and TRAF homology domain) is found in cytosolic signaling molecules such as TRAF class proteins, which are characterized by a C-terminal region encompassing about 180 amino acids, forming a 7-8 anti-parallel β-sheets fold (TRAF-C domain / MATH domain) [3-5], and the domain forms a new, light-stranded antiparallel beta sandwich structure [6]. A coiled-coil region adjacent to the MATH domain is important for oligomerisation, essential for establishing appropriate connections to form signalling complexes with TNF receptor-1 [7]. The ligand binding surface of TRAF proteins is located in beta-strands 6 and 7, and consensus motif (P/S/A/T)x (Q/E)E is the major motif of MATH domain [8]. The MATHdomain seems to be important for protein-protein interaction and several studies on human and *C. elegans* MATH proteins indicate that they might have important functions in the regulation of protein processing [9]. In TRAF proteins, the N-terminal of the MATH domain has been shown to be necessary and sufficient for self-association (homodimerization) and receptor interaction [6, 10-14].

Many MATH domain proteins that are found in humans [15-17] and mammals [2, 8, 18] are mainly involved in individual development and cell growth, differentiation, and aging as molecular adaptors. For example, TRAF6 is a critical factor for dendritic cell maturation and development [19-21]. Recently, this type of MATH proteins are also found in plants (*Arabidopsis,* Medicago, rice), in lower eukaryotes (Trypanosoma, Plasmodium) and in lower metazoa (*C. elegans*) [22].

MATH domain containing proteins are found usually associated to a discrete set of other protein domains, including peptidases, filamin and RluA domains, broad-complex, tramtrack and brie-a-brae (BTB) domain, tripartite motif (TRIM), astacin domain and RING and Zinc finger domains [9]. The BTB domain is an evolutionarily conserved domain broadly distributed in eukaryotes [23, 24]. At least 76 BTB-domain proteins exist in *Arabidopsis* belonging to 11 major families [25]. Proteins carrying both BTB and MATH motifs are common in plants. For example, there are at least 74 likely functional *MATH-BTB* genes in rice, but also at least another 40 *MATH-BTB* pseudogenes [26], whereas, only six members (AtBPM1~6) are annotated in Arabidopsis [27-29]. The MATH domain of BPM (BTB/POZ-MATH) proteins is used to assemble with members of the ethylene response factor⁄Apetala2 (ERF/AP2) transcription factor class [30]. In addition, MATH-BTB proteins may directly interact with and target the homeobox-leucine zipper (HD-ZIP) transcription factor ATHB6 (*Arabidopsis thaliana* homeobox gene 6) for proteasomal degradation [31].

Brassicaceae-specific PSV-embedded proteins (BPEPs) have an N-terminal signal peptide and two tandem MATH domains and are localized in PSVs (protein storage vacuole), and at least one BPEP is tightly associated with the phytate contained within PSV globoids [32]. Additionally, six MATH domain proteins were identified by SSH in Brassica napus, among them four proteins are involved in lipin metabolism and two proteins are related to sugar, and all have two MATH domains [33]. 

Phylogenetic analysis of MATH domain proteins has proven helpful as a guide for genetic and molecular studies of this large family of proteins. Zapata and colleagues reported that MATH domain proteins can be divided into the USP7 family, the MATHd-only family, the MATHd/BTB family and the MATHd/Filament family in Brassica, but rice lacks the MATHd/Filament family; and the TRAF, TRIM37 and Meprin families are only found in animals [9]. Phylogenetic and domain organization analysis may infer to putative functions, for example, the USP7 class containing a UBP domain at the C-terminal side of MATH domains has ubiquitin proteases (UBPs) activity [9].

Here, we identified and analyzed MATH-domain proteins from *Brassica rapa* whole genome sequence [34], together with most of* Arabidopsis thaliana*, *Oryza sativa* and *Ostreococcus tauri *[35-37], and also some from animals whose functions are known [9]. Our data provide a platform for future functional characterization of these genes in *Brassica *species. 

## MATERIALS AND METHODS

### MATH Domain Protein Identification

Sequences of MATH domain proteins from *A. thaliana* were retrieved from the TAIR Database (http://www.arabi dopsis.org, version: 2.2.8) with the key word MATH in species database. Using the *Arabidopsis thaliana* sequences of MATH proteins, we performed a search in the *O. sativa *using the BLASTp algorithm in the NCBI browser (http:// blast.ncbi.nlm.nih.gov/Blast.cgi). Then these sequences, primarily those from *A. thaliana *and *O. sativa*, were used as queries to search Brassica Database (http://brassicadb.org/brad/searchAll.php, version: 1.1) and *Ostreococcus tauri *database (http://genome.jgi-psf.org/Ostta4/Ostta4.home.html, version: 2.0) by using the BLASTp and tBLASTn tools (http://blast.ncbi.nlm.nih.gov). The Expect threshold was set at 1.0 and other parameters were set at default values.

### Protein Domain Organization Analysis

The protein sequences were analyzed for domain organization using NCBI-CD searches (http://ncbi.nlm.nih.gov/ Structure/cdd/wrpsb.cgi). The low-complexity filter was turned off, and the Expect value was set at 1.0 to detect short domains or regions of less conservation in this analysis. Domains were also verified and named according to the SMART database (http://smart.embl-heidelberg.de/).

### Phylogenetic Analysis

Multiple sequence alignments of MATH-domain proteins sequences were performed using the Clustal W program [38]. The full-length proteins were subjected to phylogenic analysis using the MEGA5.1 program [39]. The trees were constructed with the following settings: Tree Inference as Neighbor-Joining; Include Sites as pair wise deletion option for total sequences analysis; Substitution Model: Poisson correction; and Bootstrap test of 1000 replicates for internal branch reliability [40].

### qRT–PCR Analysis

*B. rapa* plants were grown at 18-22°C under a 12h light (10,000 Lx)/12h dark photoperiod. Leaves were collected from 20, 30, 35 days-old plants after artificial pollination; roots, stems and flower buds were collected from blossoming plants. Total RNA was extracted using Trizol Reagent (Invitrogen, USA) from about 100 mg of collected plant tissue. The RNA preparation was then treated with DNaseı (Promega, USA) for 30 min at 37°C, followed by enzyme inactivation by incubation at 65(C for 5min. First strand cDNA was made using an RT-PCR Kit (RevertAid™ First Strand cDNA Synthesis Kit, Fermentas, CA). The RT-solution with first strand cDNA was stored at -80°C [40]. Fluorescence-based quantitative-PCR was performed used SYBR Premix Ex Tag Reagent (Takara, Japan). Primers used for Q-PCR reactions are listed in (Table **1**). Conditions for the Q-PCR reactions were as follows: 95°C for 10 min; then 40 cycles of 95°C for 15 s, 60°C for 30 s, and 72°C for 30s (AB, USA).

## RESULTS

### Identification and Annotation of MATH-Domain Proteins from the *Brassica rapa *Genome

Using BLASTp and tBLASTn bioinformatic tools, we identified 90 genes encoding different MATH-domain proteins from the *B. rapa* genome (http://brassicadb.org/brad, Table **2**) compared to 63 genes in Arabidopsis (NCBI), 36 genes in rice (NCBI), only 2 genes in* O. tauri *database (http://genome.jgi-psf.org) and 16 genes from different animals (Table **2**, Supplement **1**). There are ten chromosomes in *Brassica rapa*, just twice to *Arabidopsis*. It suggested that genes encoding MATH domain proteins duplicated or lost during chromosome duplication. Those genes disperse to all ten chromosomes in *B. rapa* (Table **2**), except the localization of Bra029646, Bra040903 and Bra040904 are still be uncertain. Ch3 and Chr9 have much more MATH domain encoding genes than the others, and both have 16 genes (Table **2**).

### Phylogenetic Analysis of MATH-Domain Proteins

**Fig. (1):**
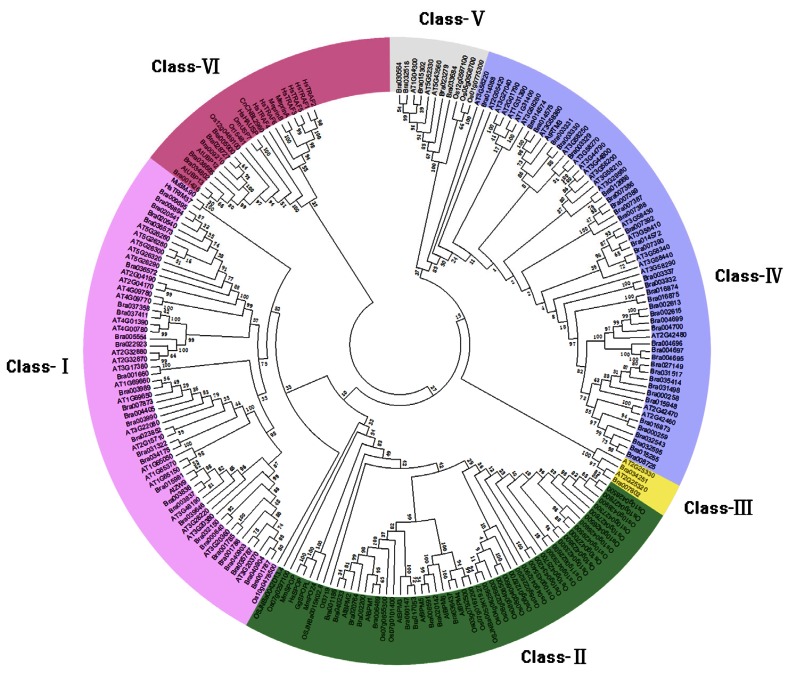
Phylogenetic tree of MATH-domain proteins in *Brassica rapa*. The MATH domain sequences of the different 185 proteins (90 proteins in *B.rapa*, 63 proteins in *Arabidopsis*, 36 proteins in rice, 2 proteins in *O.tauris*, 2 proteins in mouse, 8 proteins in human, one in bee, chicken and *Drosophila melanogaster* were aligned using Clustal W, and the phylogenetic tree analysis was performed using MEGA4.0. The trees were constructed with the following settings: Tree Inference as Neighbor-Joining; Include Sites as pairwise deletion option for total sequences analysis; Substitution Model: Poisson correction; and Bootstrap test of 1000 replicates for internal branch reliability. Supplement 2 is the tree based on maximum likelihood, and both trees are much similar.

**Fig. (2):**
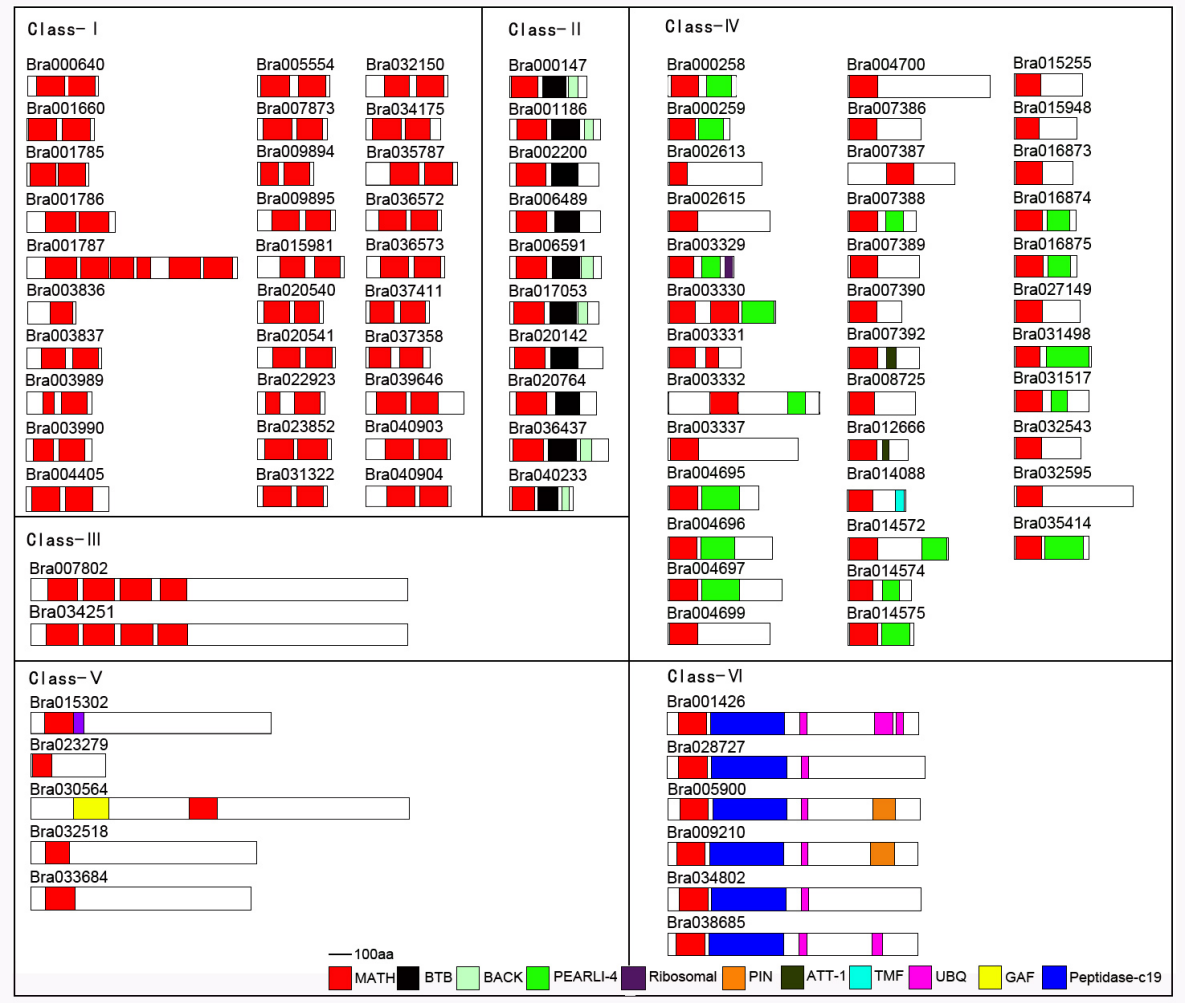
Domain architecture of the different classes of *Brassica rapa* MATH domain proteins. Domains were identified using the Conserved Domain Search service (http://ncbi.nlm.nih.gov/Structure/cdd/wrpsb.cgi) and verified and named according to the SMART database (http://smart.embl-heidelberg.de/). The low-complexity filter was turned off, and the Expect value was set at 1.0 to detect short domains or regions of less conservation in this analysis. Class-I only had two tandem MATH domain except Bra001787; Class-II had MATH and BTB domain; Class-III had Filament domain at the C-terminus in *Arabidopsis* [9], but this domain couldn’t be detected in *B.rapa*; Class-IV had one or two MATH domains, in addition to that, some proteins also has a pearl1-4 domain; Class- proteins were longer than others, and Classmembers had MATH, UBQ and Peptidase domain.

To examine the phylogenetic relationships among* B. rapa* MATH-domain proteins and group them within the established classes, we subtracted all MATH domain proteins from several species, including 63 proteins from* A. thaliana*, 36 proteins from *O. sativa,* 2 proteins from* O. tauri*, We also included the TRAF1~6 proteins, TRIM37, meprin A and B, SPOP and HAUSP from Human, MuBM-90, MmSPOP and MmPOZ4 from Mouse, SPOPL from* Gallus gallus*, BL2960 from *Cryptococcus neoformans* and USP7 from *Drosophila melanogaster*. According to the latest report, proteins encompassing MATH domains and their association with other protein domains are grouped in 8 families, and 4 families in Brassica [9]. However, based on our analysis, MATH domain proteins in *B.rapa* were divided into 6 classes, and have not TRAF and Meprin family mumbers (Fig. **1**, Fig. **2** and Supplement **2**). The last 3 classes belong to a branches group in the tree, but had much difference in domain organization.

### Conserved Domains in MATH-Domain Proteins

Additionally, we analyzed the architecture of 90 Brassica MATH domain proteins and found that they can be classified into six families based on existence of the other conserved domains combined with sequence similarity (Fig. **2**). Class-Ⅰ, including 30 MATH domain proteins, only has two tandem MATH domains except Bra001787 whose domain organization is similar to Class-Ⅲ including longer sequence and more MATH domains. But alignment results showed that Bra001787 is much similar to Class-I member (Supplement **3**), and 74% amino acid identity with Bra040904, 25% and 27% sequence identities with Class-III member Bra007802 and Bra034251 which also have 4 MATH domains, respectively. Therefore, we classified it into Class-Ⅰ, suggesting that Bra001787 might originate from Bra040904 by duplication of MATH domain tandem. All the 10 members of Class-Ⅱ have MATH and BTB domain, and some also have another BACK domain in the C-terminus. Class-Ⅲ was fallen into a branch which *Arabidopsis* proteins had Filament domain at the C-terminus [9], but this domain couldn’t be detected in *B. rapa *and *Arabidopsis* in NCBI and SMART. Class-Ⅳ contains 37 members, and is the largest class. Besides the MATH domains, some proteins have another pearl1-4 domain and Bra003329 has another Ribosomal domain in the C-terminus. But in this class, 35 proteins only contained one MATH domain. In rice, MATH only domain class is the largest family and contains about 50% MATH domain proteins [26]. Taken together, MATH only proteins have the largest members. Class-Ⅴ proteins are larger than the members in Class-I to IV except that Bra023279 only have a MATH domain. Both Class-Ⅳ and Class-Ⅴ classes belonging to same branch (Fig. **1**) and having similar MATH domain may have some uncertain relation. Class-Ⅵ numbers are also longer than first four classes and all have MATH, UBQ and Peptidase domains. Previous researches suggested that the last 2 classes have ubiquitin proteases (UBPs) activity and may be derived from a common ancestor, but some sequences lacked the ubiquitin protease domain during evolutionary process [9].

### Expression Analyses of MATH Proteins Encoding Genes in Seed Development

**Fig. (3):**
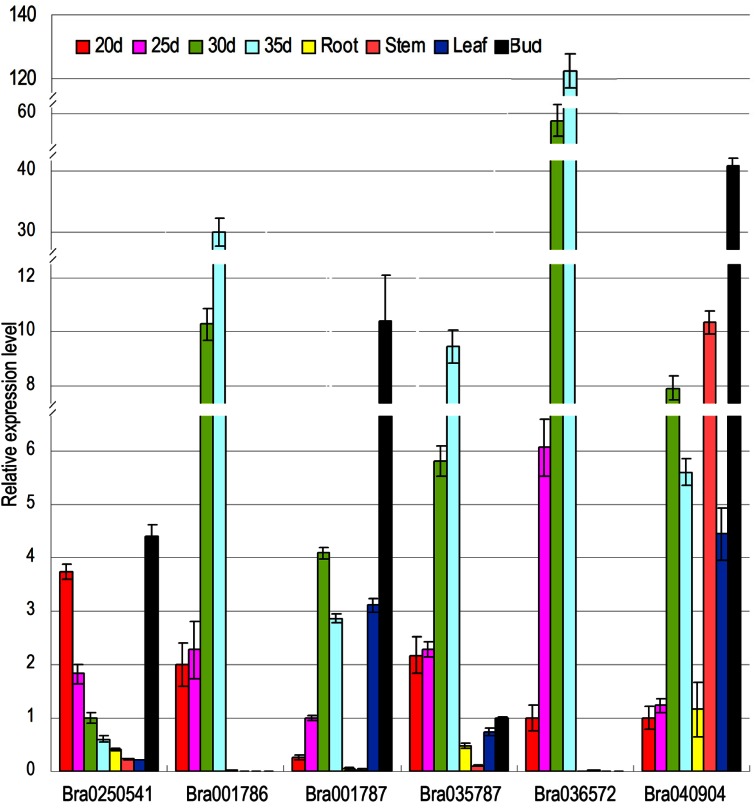
The expression of encoding MATH domain proteins genes involved in seed development. Expression pattern suggest that *Bra001786, Bra03578* and Bra036572 may be seed development special genes, but *Bra001787, Bra020541* and *Bra040904* may involve in seed and flower organ development. Material: seed, from 20, 25, 30, 35 days young seed after artificial pollination; Roots, from principal root of 15 days seedling; Stems, from young stem of 15 days seedling; Leaf, from young leaf of 15 days seedling; Bud: from young flower bud, length 􀀁 0.5cm.

SSH libraries from 20 /30 days seed development of *Brassica napus* were constructed [33], and *Bra020541* is homolog to EST T200139, which was screened in the 20-day-old seed SSH library, and was weakly expressed in root, stem, and leaf, but highly in young flower bud. During seed development, the expression level decreased more dramatically in seed at the 20-day than that at 35-day. *Bra001786*, *Bra001787*, *Bra040904* and *Bra035787* are all homolog to EST *T350008*, and *Bra036572* is homolog to EST *T350054* in the 30-day-old seed SSH library, respectively [33]. Expression of* Bra001786 *or *Bra036572 *was only detected in seed and gradually increased during seed development. *Bra001787 *had stronger expression in leaf and flower bud than in seed, and no expression in root and stem, and the expression level in seed development reached the peak at 30 days. *Bra035787* was highly expressed in root, leaf, flower bud and seed, but less in stem, and the expression level increased with seed development process. *Bra040904* was expressed in all detected organs, and much higher in flower bud and stem than in root and leaf, and the expression level increased and reached the top in seed at 30 days during seed development (Fig. **3**). Among those genes, *Bra036572* expressed much higher in seed at 30 and 35 days than other genes, especially *Bra020541*. 

According to expression of genes in seed development process, there are three expression patterns. *Bra020541 *expresses in early stage of seed development. *Bra001786* and *Bra036572* have much similar expression pattern: the expression level increases with seed development process, reaches the top in seed at 35 days, and undetectable expression in root, stem and flower bud. Similarly, *Bra03578 *expresses much higher in seed than other organs, and also reaches the top in seed at 35days, suggesting that *Bra001786*, *Bra036572* and *Bra03578 *are late developmental stage seed genes. *Bra001787* and* Bra040904 *both express higher in seed at 30 days, suggesting that they may be the regulation genes at middle stage of seed development.

## DISCUSSION

MATH domains seem to be very important for the regulation of protein processing [5, 6, 9-13]. Except class-Ⅰand class-Ⅲ, all have additional domains, such as BTB, UBQ, BACK, and GAF domains (Fig. **2**). The BTB domain also known as the POZ (for Pox virus and Zinc finger) domain [41], is an evolutionarily conserved domain broadly distributed in eukaryotes [23, 24]. Proteins carrying both BTB and MATH motifs are common in plants [23, 24, 41, 42]. Arabidopsis has 6 numbers AtBMP1-AtBMP6, but Brassica has 10 numbers (Fig. **1**), and rice has more than 30 numbers. It suggested that those genes divided and evolved after speciation. Ubiquitin domain proteins (UDPs) and ubiquitin-like (UBL) domain proteins belong to a diverse group of proteins which are characterized by an integral UBQ or UBL domain. The majority of UDPs described so far are components of the ubiquitin system which is crucial for the degradation of most cellular proteins [43, 44], In Arabidopsis, the EVE1 protein containing a 52 amino acid ubiquitin domain (UBQ) is involved in the control of inflorescence stem formation [45], and so far 7 UBQ domain proteins have been identified (Fig. **1** and Fig. **2**).

Previous reports have shown that Brassica MATH proteins fall into four classes [9], but we suppose that six classes may be more reasonable according to phylogenetic analysis and specific domain architectures in the whole genome level. Class-Ⅰ MATH-domain-only proteins is a large number of hypothetical proteins containing multiple MATH-domains in tandem except only one in Bra003836. Proteins encompassing a MATH domain and a BTB/POZ domain are broadly represented in eukaryotes (Class-Ⅱ). Many reports showed MATH-BTB proteins interacted with CUL-3 carried had E3 ubiquitin ligase activity [26, 31, 46, 47], and MAB1 exprsssed in the germ lineages and the zygote of maize [48]. Here, ten MATH-BTB domain proteins present in *B.rapa,* and conserved domain organization suggests that they might have similar function. Comparisons of sequence similarity and synteny of *B. rapa *and *A. thaliana *MATH-domain proteins revealed occurrence of recent gene duplication events (http://brassicadb.org/brad/searchSynteny.php). Bra00200, Bra006489 and Bra023700 show syntheny to AtBPM1, but the putative protein of Bra023700 has 170 aa without MATH domain, suggesting that it may shift its function during the evolution, and also fails to be clustered with others; Brao20764, Bra040233 and Bra001186 show syntheny to AtBPM2; Both Bra017053 and Bra000147 show syntheny to AtBPM3; Bra036437 show syntheny to AtBPM4; Bra 006591 and Bra020142 show syntheny to AtBPM5, but no sequence show syntheny to AtBPM6. It suggests MATH-BTB genes diploidization or triploidization or losing in *B. rapa*. Class-Ⅲ only has two Brassica proteins and two Arabidopsis proteins, and previous research showed that Arabidopsis proteins had FILAMENT domain, but they can’t be detected here. Ubiquitin Proteases (UBPs) are also found in plants (*Arabidopsis, Oryza*) and in Metazoa [9]. Except that Bra003330 and Bra003331, Class-Ⅳ proteins have only one MATH domain, and about half of them as well as have unknown function PEARLI-4 domain (Arabidopsis phospholipase-like protein). AtRTM3 belonging to Class-Ⅳ, is the first biological function identified in a resistance mechanism in plant, encodes a MATH and CC domain protein [5], and restricts plant viruses long distance movement [49], and its syntheny gene Bra014574 may have similar function. All MATH domains in Class-Ⅴ are localized at the N-terminal except Bra030564 in the middle. Interestingly, Class-Ⅵ has 7 putative proteins with UBQ domain which have ubiquitin proteases (UBPs) activity and Peptidase-C19 domain.

MATH domain proteins are found as cytosolic signaling molecules in animals [3]. Less plant MATH domain proteins are identified, such as, RTM3 (belong to Class-IV) can restricts plant viruses long distance movement [5, 49], and MATH–BTB domain proteins (Class-II) directly interact with and target transcription factor ATHB6 for proteasomal degradation [31]. Our previous research showed that 6 MATH domain proteins are involved in seed development of *Brassica nupas* [33], here 6 *B.rapa *homologous genes Bra001786, Bra001787, Bra020541, Bra036572, Bra035787 and Bra040904 are identified and all have only MATH domain and belong to Class-Ⅰ. The expression of *Bra020541*, homolog to EST *T200139* in 20-day-old seed SSH library, *Bra001786*, *Bra001787*, *Bra040904*, *Bra035787*, homolog to EST *T350008* and *Bra036572*, homolog to EST *T350054* in 35-day-old seed SSH library [33]. The expression pattern suggest that* Bra001786*, *Bra03578* and Bra036572 may be seed development specific genes, but *Bra001787*, *Bra 020541* and *Bra040904* may be involved in seed and flower organ development, indicating that MATH domain proteins may have common and/or separate functions during the evolution.

## Figures and Tables

**Table 1.** qRT-PCR Primers for MATH Domain Protein Encoding Genes Involved in Seed Development.

Gene	Forward	Reverse
Bra001786	CAATCCCTCATACGACAATCCA	GTCACTTGGTTTGAAGGTGAGA
Bra001787	GGAGTTTCTCCGCGTTGGT	GCACGGCCACTTGGATTTAC
Bra035787	GTTGGGAAACTGGACGTGCTT	CCACAAGAAACCCCTTTGATG
Bra040904	CCAATGTGTGGCGTTTTAATGT	ATCCGTTTGCCGGGTCTT
Bra036572	GTTGAGAAGCGAGCGACCAT	ACTGAGGATTCGTACGGTTCAA
Bra020541	TCCCTTTGGTTGGGAACTCA	GCCCCATCTGTGACAGTCAA

**Table 2.** MATH Domain Proteins in *Brassica rapa*.

Name	Arabidopsis Synteny	ORF Length (bp)	Protein Length (aa)	Genomic Length (bp)	No. of Introns	Chromosome
**Class-I**						
Bra000640	AT4G09770	963	320	1460	4	3
Bra001660	AT3G17380	864	287	1345	4	3
Bra001785	AT3G20360	849	282	1499	4	3
Bra001786	AT3G20360	1200	399	10892	6	3
Bra001787	AT3G20360	3993	1330	10191	14	3
Bra003836	UN	684	227	1481	2	7
Bra003837	UN	1014	337	1660	4	7
Bra003989	UN	873	290	1873	2	7
Bra003990	AT1G69650	903	300	1407	4	7
Bra004405	AT1G69650	1101	366	1666	5	7
Bra005554	AT2G32870	927	308	1565	5	5
Bra007873	AT1G69650	948	315	1308	4	2
Bra009894	AT5G26280	768	255	1216	4	6
Bra009895	AT5G26280	1053	350	1705	6	6
Bra015981	UN	963	382	1783	4	7
Bra020540	AT5G26280	900	299	1921	5	2
Bra020541	AT5G26280	1056	351	1886	5	2
Bra022923	AT2G32870	921	306	1865	5	3
Bra023852	AT3G22080	963	320	1509	4	1
Bra031322	AT3G22080	933	310	1285	4	5
Bra032150	UN	1074	357	1506	4	4
Bra034175	UN	1005	334	3167	1	1
Bra035787	AT3G20370	1215	404	1694	5	5
Bra036572	AT5G26280	1035	344	1753	6	9
Bra036573	AT5G26280	1053	350	1864	5	9
Bra037358	AT4G00780	867	288	1269	4	9
Bra037411	AT4G01390	861	286	1225	4	9
Bra039646	UN	1332	443	2936	5	Scaffold000172
Bra040903	UN	1149	382	1862	5	Scaffold000303
Bra040904	UN	1149	382	1813	5	Scaffold000303
**Class-II**						
Bra000147	AT2G39760	1149	382	2759	3	3
Bra001186	AT3G06190	1212	403	1815	3	3
Bra002200	AT5G19000	1203	400	2348	3	10
Bra006489	AT5G19000	1212	403	1969	3	3
**Class-II**						
Bra006591	AT5G21010	1233	410	2144	3	3
Bra017053	AT2G39760	1200	399	1941	3	4
Bra020142	AT5G21010	1248	415	2220	3	2
Bra020764	AT3G06190	1158	385	1819	3	2
Bra036437	AT3G03740	1341	446	2703	3	1
Bra040233	AT3G06190	1170	389	2174	3	7
**Class-III**						
Bra007802	AT2G25320	4995	1664	5968	9	9
Bra034251	AT2G25320	4944	1647	5945	9	4
**Class-IV**						
Bra000258	AT2G42460	924	307	1861	4	3
Bra000259	AT2G42460	861	286	1335	3	3
Bra002613	UN	1257	418	1636	3	10
Bra002615	UN	1347	448	1665	3	10
Bra003329	AT3G58230	885	294	1595	4	7
Bra003330	AT3G58230	1440	479	6302	7	7
Bra003331	AT3G58230	981	326	3134	5	7
Bra003332	AT3G58230	2016	671	2659	5	7
Bra003337	AT3G58400	1740	579	4083	5	7
Bra004695	AT2G42460	1215	404	1593	3	5
Bra004696	AT2G42460	1392	463	1778	3	5
Bra004697	AT2G42460	1518	505	2209	4	5
Bra004699	AT2G42460	1371	456	1848	3	5
Bra004700	AT2G42460	1896	631	2301	2	5
Bra007386	AT3G58200	981	326	1319	3	9
Bra007387	AT3G58200	1416	471	2662	6	9
Bra007388	AT3G58200	915	304	1317	3	9
Bra007389	AT3G58200	960	319	1302	3	9
Bra007390	AT3G58200	741	246	1077	3	9
Bra007392	AT3G58200	981	326	1393	3	9
Bra008725	UN	909	302	1209	3	10
Bra012666	UN	819	272	1252	3	3
Bra014088	UN	810	269	1859	4	8
Bra014572	AT3G58400	1347	448	2885	4	4
Bra014574	AT3G58320	855	284	1670	3	4
**Class-IV**						
Bra014575	AT3G58320	870	289	1538	3	4
Bra015255	UN	909	302	1198	3	10
Bra015948	UN	843	280	1208	3	7
Bra016873	AT2G42460	813	270	1281	3	4
Bra016874	AT2G42460	840	279	1078	2	4
Bra016875	AT2G42460	858	285	1034	2	4
Bra027149	UN	888	295	1209	3	9
Bra031498	UN	1062	353	1459	3	1
Bra031517	UN	1017	338	1287	3	1
Bra032543	UN	831	276	1207	4	9
Bra032595	UN	1623	540	2136	5	9
Bra035414	UN	1071	356	1559	3	1
**Class-V**						
Bra015302	AT1G04300	3183	1060	4995	12	10
Bra023279	UN	1050	349	1860	6	9
Bra030564	AT1G04300	5052	1683	8328	13	8
Bra032518	AT1G04300	2985	994	4568	10	9
Bra033684	AT5G43560	2784	927	4176	11	6
**Class-VI**						
Bra001426	AT3G11910	3339	1112	8265	32	3
Bra005900	AT5G06600	3348	1115	8460	32	3
Bra009210	AT5G06600	3303	1100	7823	31	3
Bra028727	AT5G06600	3417	1138	8289	32	2
Bra034802	AT3G11910	3348	1115	8732	32	5
Bra038685	AT3G11910	3309	1102	7657	31	1

Data coming from web http://brassicadb.org/brad/. UN=uncertainly.
